# Is hypercalcaemia immediately life-threatening? A prospective study

**DOI:** 10.1530/EC-24-0508

**Published:** 2025-01-27

**Authors:** David Trewick, Mathilde Le Borgne, Julie Regnault, Camille Guimard

**Affiliations:** ^1^ Department of Medicine, CHD Montaigu, Montaigu, France; ^2^ Department of Emergency Medicine, Nantes University, Nantes, France; ^3^ Department of Medicine, Clinique Jules Verne, Nantes, France

**Keywords:** hypercalcaemia, coma, life-threatening arrhythmias, QT interval

## Abstract

**Objective:**

Hypercalcaemia is often considered as an emergency because of a potential risk of life-threatening arrhythmias or coma. However, there is little evidence, apart from case studies, that hypercalcaemia can be immediately life-threatening. The aim of our study was to prospectively assess whether hypercalcaemia (Ca ≥ 3 mmol/L) was associated with immediately life-threatening complications.

**Design and methods:**

We conducted a prospective observational study aiming to include the first one hundred patients aged ≥18 who had a calcium concentration ≥3 mmol/L, admitted to the emergency department (ED). The primary outcome was the number of life-threatening cardiac arrhythmias (ventricular tachycardia, ventricular fibrillation, sinus arrest and second- or third-degree atrioventricular blocks) or neurological complications defined by a Glasgow Coma Scale score <9 during the stay in the ED. The secondary outcomes were correlation between calcium concentrations and ECG (electrocardiogram) QTc intervals, Glasgow Coma Scale scores and mortality during the following 12-month follow-up period.

**Results:**

The median calcium concentration was 3.3 mmol/L (3.1–3.7). Cancer was the first cause of hypercalcaemia. No patient presented a life-threatening cardiac arrhythmia during their stay in the ED. Three patients presented a life-threatening neurological complication. There was no correlation between calcaemia and QTc intervals or Glasgow Coma Scale score. Prognosis was poor, and 43 patients died during the 12 months.

**Conclusions:**

We found no cases of immediately life-threatening cardiac arrhythmias. Three patients had indeed a life-threatening neurological complication but always had at least one other major factor that could severely alter mental status, such as profound metabolic acidosis.

**Significance statement:**

This paper aims to revisit what most physicians, whether specialists or not, consider to be scientifically proven facts concerning the immediate threat caused by hypercalcaemia. Its novelty is threefold: first, this is the only prospective study that exists to date studying the life-threatening consequences of hypercalcaemia; second, having included one hundred patients, we found no life-threatening cardiac arrhythmias, which is not what would be expected if one reads guidelines concerning hypercalcaemia; and third, life-threatening neurological complications were very rare and only occurred in patients with at least one other major cause of altered neurological status, such as severe metabolic acidosis or hypernatraemia.

## Introduction

Hypercalcaemia is often considered as an emergency because of a potential risk of life-threatening arrhythmias, such as ventricular tachycardia (VT), ventricular fibrillation (VF), sinus arrest and second-degree or third-degree atrioventricular blocks, or life-threatening neurological complications, such as coma (1, 2, 3, 4). These assertions are anchored in our minds and textbooks, are tirelessly repeated to our students and are considered by many to be an essential part of the fundamental basic knowledge that one requires to deal with metabolic emergencies. However, there is little evidence that hypercalcaemia can be immediately life-threatening. Our knowledge of the subject and our guidelines are based on few and often very unconvincing case reports, which often fail to report or simply neglect the role of other electrolytes, such as potassium, or the presence of underlying heart disease and heart medication (5, 6, 7, 8). In other cases, severe symptoms are attributed to very mild cases of hypercalcaemia, in fact almost normal levels of calcium (8). New guidelines make references to older guidelines, without first revisiting the original papers (1, 2, 3, 4).

We were the first to specifically investigate the immediate threat posed by severe hypercalcaemia. We retrospectively investigated 31 patients with very high calcium concentrations, in excess of 4 mmol/L (16 mg/dL), and found no cases of life-threatening arrhythmias or life-threatening neurological complications (9).

The aim of this study was to prospectively assess whether hypercalcaemia (Ca ≥ 3 mmol/L) was associated with immediately life-threatening cardiac arrhythmias or neurological complications in patients admitted to the emergency department (ED).

## Materials and methods

We conducted a prospective observational study aiming to include the first one hundred patients aged ≥18 who had a calcium concentration ≥3 mmol/L, admitted to the adult ED of Nantes University Hospital (86,000 annual visits). Inclusions started on the 1 March 2019 and finished in May 2021.

Blood samples were collected and centrifuged at 2000 *g* for 10 min at 4°C within 1 h after venipuncture. All biochemical measurements of calcium were performed in the same laboratory (Laboratory of Clinical Biochemistry, University Hospital of Nantes) with a photometric Calcium Gen.2 assay based on photometric measurement of the calcium-NM-BAPTA complex on Cobas c701 (Roche Diagnostics, Germany) according to the manufacturer’s instructions. Albumin-corrected calcium levels were estimated as described previously using the following equation: corrected calcium = measured calcium (mmol/L) + 0.02 [40-albumin (g/L)] (10).

Patients’ records were reviewed, and relevant clinical and biological data were collected. The primary outcome was the number of life-threatening cardiac arrhythmias and/or neurological complications during the stay in the ED. A life-threatening cardiac arrhythmia was defined by the presence of VT, VF, sinus arrest and second-degree or third-degree atrioventricular blocks. A life-threatening neurological condition was defined by the presence of a coma with a Glasgow Coma Scale score (GCS) of less than 9/15 (11). The secondary outcomes were correlation between calcium concentrations and ECG QTc intervals/GCS and mortality during the 12-months follow-up period.

ECGs were blindly interpreted by two of the authors (CG and DT). Methods for measuring the corrected QT intervals are described in detail elsewhere (12). Normal QTc intervals were 360–450 ms for males and 370–470 ms for females.

Ethical approval was obtained from the Ethics Committee of the Nantes University Hospital. The trial was recorded under the number RNI_0062-IM-193-V8. Written informed consent was obtained from all patients or legal guardians or the patient’s next of kin when neurological status prohibited consent.

All data were presented as median and first (Q1) and third (Q3) quartiles. Correlation was tested using Spearman’s method, and means were compared using paired *t*-tests. A *P*-value less than 0.05 was considered statistically significant. Survival analysis was performed using the Kaplan–Meier regression model.

## Results

### Patient selection

The first 100 patients with calcium ≥ 3 mmol/L were included. No patient was excluded. A total of 52,972 adult patients admitted to the ED had calcium concentrations measured over the study period. The incidence of hypercalcaemia as defined above was 0.19%.

### Patients’ characteristics

The median calcium concentration was 3.3 mmol/L (3.1–3.7), the median albumin-adjusted calcium concentration was 3.3 mmol/L (3.1–3.6), and 34 patients had severe hypercalcaemia (Ca > 3.5 mmol/L). Patients’ characteristics during the stay in the ED are presented in Table 1. In addition, there were no cases of pancreatitis or seizures and three patients had focal neurological signs, two of which were attributable to intracranial lesions (ischaemic stroke and cerebral venous infarction with haemorrhagic transformation) and the third was attributed to paraneoplastic cranial nerve involvement. No focal sign regressed upon correction of hypercalcaemia. Table 2 shows the relevant biological data, and Table 3 details the causes of hypercalcaemia. Cancer was the first cause of hypercalcaemia (59 patients), with the most frequent being lung cancer (14 patients), followed by multiple myeloma, breast cancer and kidney cancer. Hypercalcaemia led to the discovery of an unknown cancer in 15 patients. Primary hyperparathyroidism was the second most frequent cause (23 patients).

**Table 1:** Patients’ characteristics (*n* = 100).

Patients’ characteristics	*n*
**Baseline characteristics**	
Age (years)	72 (Q1 60 Q3 82)
Female	53
Cardioactive drugs*	19
Sedatives and opioid analgesics	17
Hypertension	48
Ischaemic heart disease	11
Previous history of cancer or cancer currently under treatment	56
Dementia	8
**Clinical features**	
Mean systolic BP (mmHg)	146 ± 20
Mean diastolic BP (mmHg)	76 ± 14
Mean heart rate (bpm)	91 ± 16
GCS score	
GCS = 15	68
GCS = 14	25
GCS between 9 and 14	4
GCS < 9	3
Nausea/vomiting	30
Abdominal pain	25
Hypertension	42

BP, blood pressure; GCS, Glasgow Coma Scale score.

* β-Blockers, digoxin and non-dihydropyridine calcium channel blockers.

**Table 2:** Biological data (*n* = 100).

Biological data	Median	Q1	Q3	IQR	Range
Calcium (mmol/L)	3.3	3.1	3.7	0.4	3–5.2
Corrected calcium* (mmol/L)	3.3	3.1	3.6	0.4	3–4.4
Phosphate (mmol/L)	0.9	0.7	1.1	0.3	0.3–3.8
Sodium (mmol/L)	138	135	141	4.2	128–163
Potassium (mmol/L)	4	3.6	4.5	0.6	2–7.8
BUN (mmol/L)	10.9	7.5	15	5.1	2.8–53.7
Creatinine (μmol/L)	114	77	177	71	36–1152

BUN, blood urea nitrogen.

* Albumin-corrected total calcium.

**Table 3:** Causes of hypercalcaemia (*n* = 100).

Causes of hypercalcaemia	*n*
Malignancy	59
Solid tumours	45
Haematological malignancies	14
Primary hyperparathyroidism	23
Alfacalcidol intoxication	6
Others	
No diagnosis	5
Sarcoidosis	3

### Life-threatening events

#### Cardiac events

No patient presented a life-threatening cardiac arrhythmia (VT, VF, sinus arrest and second-degree or third-degree atrioventricular blocks) during their stay in the ED.

#### Neurological events

Sixty-eight patients had no neurological symptoms (GCS 15), 25 patients were confused (GCS 14), four patients had a GCS between 13 and 9, and three patients presented a life-threatening neurological complication, i.e., a coma, defined by a GCS < 9. The first was a 44-year-old woman with type 1 diabetes, admitted for ketoacidotic coma. She was hypothermic (temperature 34.8°C), had hyperglycaemia in excess of 30 mmol/L and had very severe metabolic acidosis (pH 6.58). Her calcium was 3 mmol/L. The second was an 83-year-old man with laryngeal cancer, admitted for acute respiratory distress syndrome, associated with severe hypernatraemia (Na 163 mmol/L). Calcium was 3.8 mmol/L. The last was an 82-year-old woman with metastatic endometrial cancer, presenting with multiorgan failure and acute respiratory distress syndrome on admission. Calcium was 3.5 mmol/L. There was no correlation between calcium levels and GCS (*R*^2^ = −0.09). There was also no correlation between sodium levels or the use of sedative medication and GCS (respectively, *R*^2^ = −0.09 and *R*^2^ = 0.1).

### Correlation between calcium and QTc intervals

The median heart rate-corrected QT interval (QTc) was 426 ms (407–464). QTc intervals were decreased, normal and increased in 5, 11 and 84 patients, respectively. There was no correlation between calcium levels and QTc intervals (*R*^2^ = 0.02). When we excluded the 20 patients with hypokalaemia (K < 3.5 mmol/L), there was still no correlation between QTc intervals and calcium concentration (*R*^2^ = 0.02). There was no correlation between QTc intervals and the use of QTc-interval-modifying drugs, i.e., β-blockers, digoxin and non-dihydropyridine calcium channel blockers (*R*^2^ = −0.16), or between QTc intervals and previous history of hypertension or ischaemic heart disease (*R*^2^ = 0.08).

### Administered therapy on the ED

Intravenous (IV) bisphosphonates were administered in the ED within the first 24 h in 73 patients (pamidronic acid: 69 patients; zoledronic acid: four patients), including four of the six patients with alfacalcidol intoxication; fluid resuscitation with isotonic saline was undertaken for 97 patients; four patients had corticosteroids (one patient with alfacalcidol intoxication, two with haematological malignancies and one with metastatic lung cancer); and two patients had IV furosemide because of fluid overload. No patient underwent dialysis (in the ED or during follow-up).

### Outcome

No patient died while in the ED. The patients were all transferred from the ED in less than 24 h. Six patients were transferred to a critical care unit; their median calcium was 3.6 mmol/L. Most of the remaining patients were admitted to a general medical ward and no longer had continuous cardiac monitoring, only 13 were admitted to nephrology, and four were admitted to endocrinology. Three patients were directly discharged from the ED: two for palliative care at home and one was diagnosed with probable primary hyperparathyroidism, and specialised follow-up was organised.

**Figure 1:**
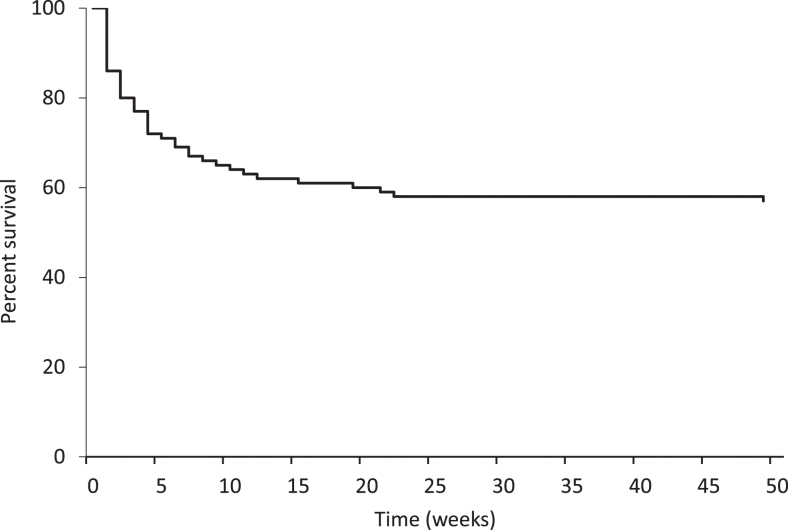
Regression analysis of survival during the 12 months.

**Figure 2:**
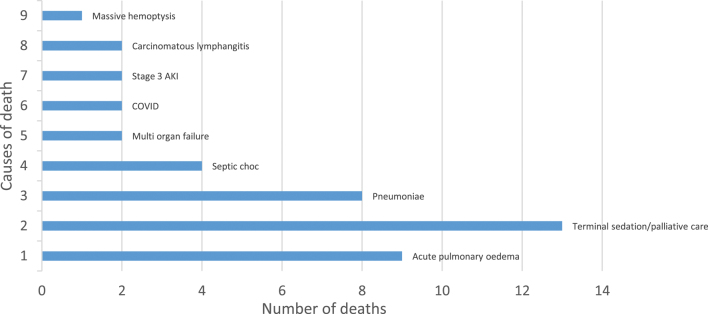
Causes of death during the 12 months.

Prognosis was extremely poor, with 43 patients dying by the end of the 12-month follow-up period; most had died by 10 weeks (Fig. 1). The causes of death are listed in Fig. 2. The most frequent causes were respiratory (pneumonia; acute pulmonary oedema) and terminal sedation instated for palliative care. Calcium levels prior to death were also significantly lower than calcium levels on admission for these patients. Indeed, calcium measurements were available during the 7 days prior to death for 41 patients of the 43 that died. Comparison of the mean calcium (2.9 mmol/L) in the 7 days prior to death and the mean calcium (3.59 mmol/L) on admission to the ED for the patients who would later die showed a significant difference (*P* < 0.001).

## Discussion

This study is the first to prospectively investigate the immediate threat posed by hypercalcaemia defined by a calcium concentration ≥3 mmol/L. We found no relation between hypercalcaemia and life-threatening cardiac arrhythmias. These findings are supported by experimental animal studies (13, 14, 15). Overall, experiments on all kinds of animals infused with calcium chloride or calcium gluconate, whether large or small (guinea pigs, dogs and cattle), tend to point to the fact that very high levels of calcium (never seen in humans) are required to bring about their death. For example, guinea pigs only died when hypercalcaemia exceeded 25 mmol/L, that is 10 times the initial calcium concentration. Furthermore, these animals had also developed severe hyperkalaemia (mean potassium 7.6 mmol/L), which is a well-known cause of fatal cardiac arrhythmias (13).

In humans, fatal rhythm disturbances associated with hypercalcaemia have been reported mainly in case reports, which form the backbone of our knowledge concerning the complications of hypercalcaemia. These patients often had underlying proarrhythmogenic heart disease and were on cardioactive medication, and most had abnormal potassium (when reported), making it difficult to conclude (5, 6, 7). We were the first to specifically report on life-threatening complications associated with hypercalcaemia. Indeed, we retrospectively analysed 31 patients who were admitted to the ED, over a 5-year period, with very severe hypercalcaemia (Ca > 4 mmol/L). We found no evidence of life-threatening cardiac arrhythmias (9). In a retrospective paper studying the epidemiology, general clinical features and ECGs of 131 patients admitted to an ICU (Intensive Care Unit), with a calcium concentration >3 mmol/L, only one patient presented a life-threatening cardiac arrhythmia, which was VT. This patient also had coexisting hypokalaemia. Once again, this is not fully taken into account in the discussion and the precise potassium concentration is not detailed (16). Other studies investigating different aspects of hypercalcaemia, such as QT intervals, reported and described ECG changes (17, 18). Even though these studies were not directly aimed at searching for life-threatening cardiac events, these events were investigated and none were found.

To conclude this section, it is worth remembering the past, when physiologists reported, during the 1980s and 1990s, experimentally induced hypercalcaemia in healthy volunteers by infusion of PTH or calcium gluconate (19, 20, 21). Calcium concentrations of up to 3.25 mmol/L were obtained and sometimes maintained for up to 14 days without any reported complications, whether cardiac, neurological or psychiatric. According to current guidelines, ‘prompt treatment would be usually indicated’ for these healthy volunteers, i.e., hyperhydration and bisphosphonates (4).

Sixty-eight patients had no neurological symptoms; however, three patients were in a coma and met our definition of a life-threatening neurological complication. These patients were indeed very ill, and all presented at least one other major factor that could severely alter mental status, such as hypothermia, very severe metabolic acidosis (pH 6.58), hypernatraemia (Na 163 mmol/L), acute respiratory distress syndrome and multiorgan failure. Furthermore, we found no correlation between GCS and calcium levels. In human pathology, few cases of life-threatening neurological complications relating to hypercalcaemia have been published (22, 23). These reports do not use a proper coma scale, such as the GCS, and therefore may have overestimated life-threatening neurological complications, and they often fail to report coexistent disorders, including dysnatraemia, space-occupying lesions and the use of sedative drugs. In our retrospective study of 31 patients with very severe hypercalcaemia (Ca > 4 mmol/L), only one patient was in coma (GCS < 9) (9). This patient was an 85-year-old female with dementia, taking sedatives, who presented with acute respiratory distress syndrome and had severe coexisting hypernatraemia (160 mmol/L). Seizures and focal neurological signs are sometimes presented as possible clinical features of hypercalcaemia (24). We found no seizures, which is consistent with previous work, because hypercalcaemia is associated with reduced neuronal membrane excitability and thus should not cause epilepsy (9, 25). Only three patients had focal neurological signs, all of which could be accounted for by the presence of specific neurological conditions, such as stroke, and did not regress once hypercalcaemia was controlled. Our previous retrospective study, which included a series of patients with very severe hypercalcaemia (Ca > 4 mmol/L), found no patients with focal signs. The exact relationship between the brain and serum calcium levels is complex, and it is difficult to extrapolate on the neurological consequences of a rise in calcium. Indeed, experimental studies show that calcium concentration in the cerebrospinal fluid (CSF) and brain remains remarkably close to normal despite hypercalcaemia and it is only in the presence of acute renal failure that brain calcium concentration (but not that of CSF) increases and EEG modifications occur (26, 27, 28). Of interest, only one of the three comatose patients in our series had acute kidney failure (creatinine 170 µmol/L).

Hypercalcaemia is typically described as responsible for shortening of QTc intervals. However, we found that there was no correlation between QTc intervals and calcium levels. Interestingly, in a series of patients with mild hypercalcaemia due to primary hyperparathyroidism, there was no significant difference between QTc intervals before and after calcium levels were normalised following parathyroidectomy (29). Many other studies have reported that shortening of the QTc interval is not a frequent feature of hypercalcaemia or that calcium levels are not correlated with QTc intervals (9, 16, 29, 30). Others, however, have found that the opposite is true (17, 18). One possible explanation for the lack of consistency in shortening of the QTc interval could be associated hypokalaemia, which would tend to lengthen rather than shorten QTc intervals, or associated medication, such as beta-blockers. When we excluded the 20 patients with hypokalaemia (K < 3.5 mmol/L), there was still no correlation between QTc intervals and calcium concentration. The scientific uncertainty around shortening of the QTc interval and hypercalcaemia is now well known and should be sufficient for this to be removed from guidelines as a stated fact.

Management in our ED followed pre-existing local guidelines, which recommend fluid resuscitation and rapid introduction of bisphosphonates if Ca concentration is ≥3 mmol/L, except in patients with alfacalcidol intoxication where management consists of fluid resuscitation and stopping the offending drug. The rapid introduction of bisphosphonates takes into account that well over 50% of patients admitted to the ED with hypercalcaemia ≥3 mmol/L have cancer and that fluid resuscitation alone will probably not suffice. The exact moment at which bisphosphonates are introduced, i.e., whether in the ED or later during the hospital stay, is a matter of debate and has not been the object of specific trials. However, four patients had no reason to receive bisphosphonates but did (four of the six alfacalcidol intoxications) because the ED physician did not follow local guidelines.

Of interest, the vast majority of patients were admitted to non-specialised units, i.e., units other than endocrinology, nephrology, rheumatology or ICU. This underscores the importance of clear-cut guidelines based on prospective studies rather than case reports.

## Limitations

Our study was non-interventional, which can introduce several well-known types of bias and notably confounding bias. We tried to control this with a robust study design, which was developed following our first retrospective study of hypercalcaemic patients published in 2017. Special attention was paid to include a well-defined population, key variables and objectives were precisely defined, and covariables such as medication and dyskalaemia were systematically recorded.

In conclusion, our study was the first to prospectively investigate the immediate threat posed by hypercalcaemia defined by a calcium concentration ≥3 mmol/L in a large tertiary ED. One hundred patients were included. We found no cases of immediately life-threatening cardiac arrhythmias (VT, VF, sinus arrest and second-degree or third-degree atrioventricular blocks). Three patients (3%) did, however, present immediately life-threatening neurological complications defined by coma with a GCS < 9/15, but all had at least one other major factor that could severely alter mental status, such as hypothermia, severe metabolic acidosis, severe hypernatraemia, acute respiratory distress syndrome and multiorgan failure.

## Declaration of interest

The authors declare that there is no conflict of interest that could be perceived as prejudicing the impartiality of the research reported.

## Funding

This research did not receive any specific grant from any funding agency in the public, commercial or not-for-profit sector.

## Data availability

Data are available as excel tables on request.
